# Optical Fiber pH and Dissolved Oxygen Sensors for Bioreactor Monitoring: A Review

**DOI:** 10.3390/s26010010

**Published:** 2025-12-19

**Authors:** Guoqiang Cui, Rui Wu, Lidan Cao, Sabrina Abedin, Kanika Goel, Seongkyu Yoon, Xingwei Wang

**Affiliations:** 1 Department of Electrical and Computer Engineering, University of Massachusetts Lowell, Lowell, MA 01854, USA; guoqiang_cui@student.uml.edu (G.C.); rui_wu@student.uml.edu (R.W.); lidan_cao@student.uml.edu (L.C.); sabrina_abedin@student.uml.edu (S.A.); 2 Department of Biomedical Engineering and Biotechnology, University of Massachusetts Lowell, Lowell, MA 01854, USA; kanika_goel@student.uml.edu; 3 Department of Chemical Engineering, University of Massachusetts Lowell, Lowell, MA 01854, USA; seongkyu_yoon@uml.edu

**Keywords:** optical fiber sensor, optical fiber applications, bioreactor monitoring

## Abstract

In the bioprocessing industry, real-time monitoring of bioreactors is essential to ensuring product quality and process efficiency. Conventional monitoring methods can satisfy some needs but suffer from calibration drift, limited spatial coverage, and incompatibility with harsh or miniaturized environments. Optical fiber sensors, with their high sensitivity, remote monitoring capability, compact size, and multiplexing, have become a promising technology for in situ bioreactor monitoring. This review summarizes recent progress in optical fiber sensors for key bioreactor parameters, with an emphasis on pH and dissolved oxygen (DO), and briefly covers temperature and pressure monitoring. Different sensing mechanisms, materials, and fiber architectures are compared in terms of sensitivity, response time, stability, and integration strategies in laboratory and industrial-scale bioreactors. Finally, current challenges and future trends are discussed, including multi-parameter sensing, long-term reliability, and the integration of optical fiber sensors with process analytical technology and data-driven control for intelligent bioprocessing.

## 1. Introduction

Bioreactors are the core workhorses of modern biomanufacturing, supporting the large-scale cultivation of microorganisms and animal and plant cells to produce therapeutic proteins, vaccines, and other biologics [1,2]. To keep these cultures in a healthy and productive state, key process variables must be kept within appropriate ranges throughout the run. Among the many parameters that can be measured, pH and dissolved oxygen (DO) are universally recognized as two of the most critical, because they are directly linked to cell metabolism, energy generation, and product formation [3,4]. Small and long-lasting deviations in pH or DO may lead to shifts in growth rate, by-product formation, and product quality, or in extreme cases to culture collapse [5,6].

In most conventional setups, pH and DO are monitored by electrochemical probes inserted directly into the bioreactor. Glass electrodes are routinely used for pH measurement, while polarographic and galvanic sensors are standard tools for DO monitoring [7,8,9]. These probes are well established and are supported by commercial control systems, which have made them the default choice from laboratory reactors to production-scale stainless-steel vessels [7]. Off-line and at-line measurements with benchtop analyzers or blood-gas analyzers are commonly performed in parallel, both to cross-check inline measurements and to monitor systems where probe installation is not feasible [8]. Together, these tools form the traditional backbone of pH and DO monitoring in bioprocessing.

Despite their long history, traditional methods exhibit several weaknesses when they are used in more demanding or less conventional bioprocesses. Electrochemical probes are prone to signal drift, fouling, and sensitivity to temperature, pressure, and medium composition, which increases the need for frequent calibration and maintenance [9]. They usually provide only one measurement point per probe and can be difficult to integrate into very small, parallel, or non-standard cultivation formats, such as microbioreactors, shake flasks, or microfluidic systems [10]. Sampling-based measurements suffer from being intermittent; the resulting time lag between sampling and analysis can mask fast process dynamics and add additional handling and contamination risks [11]. As bioprocesses move toward higher cell densities, intensified operation, and increased use of small-scale development tools, the limitations of traditional monitoring tools become more apparent and motivate the search for alternative sensing approaches.

Optical fiber sensors have attracted growing attention as one such alternative. Because the sensing signal is carried by light along a thin fiber, these devices combine a very small footprint with high sensitivity, immunity to electromagnetic interference, and the possibility of remote and multiplexed measurements [12]. Over the past two decades, a variety of optical fiber pH and DO sensors have been reported, based on fluorescence, absorption, interferometric, and grating-based mechanisms; many of them have been tested in microbial and mammalian cell cultures and in different bioreactor configurations [13]. Optical fiber technology has also been extended to parameters such as temperature and pressure, making it possible to envision multi-parameter sensing platforms that can support deeper process understanding and control [14]. These developments are consistent with the goals of Process Analytical Technology (PAT) and Quality by Design (QbD), which call for real-time measurement and control of critical quality attributes to ensure robust biopharmaceutical manufacturing [15,16].

Table 1 summarizes typical growth characteristics, cultivation time scales, and operating pH and DO ranges for representative culture types used in bioprocessing, including microorganisms, plant cells, mammalian cells, insect cells, and stem cells [17]. These operating windows outline the dynamic range and performance targets that practical pH and DO sensors need to achieve. On this basis, the present review concentrates on optical fiber pH and DO sensors for bioreactor monitoring, with a brief discussion of temperature and pressure sensing. The following sections first revisit traditional monitoring methods and their limitations, then introduce the main optical fiber sensing mechanisms and architectures, and finally compare representative pH and DO fiber-optic sensors in terms of their performance and integration strategies in bioprocess applications. Current challenges and future directions, including multi-parameter sensing, long-term reliability, and the integration of optical fiber sensors within PAT frameworks and data-driven control schemes, are also discussed.

## 2. Traditional Monitoring Methods and Their Limitations

### 2.1. History and Application of Traditional Monitoring Methods

Traditional monitoring strategies for pH and DO have been shaped by decades of experience in fermentation and cell culture processes. In most laboratory and industrial bioreactors, pH is measured with glass electrodes and DO is monitored with polarographic or galvanic electrochemical probes inserted directly into the vessel [9,18]. These instruments are compatible with standard bioreactor headplates and control systems and, therefore, have become the default option for routine monitoring from bench-scale reactors to large stainless-steel units [19]. In typical stirred-tank configurations, the pH signal is used to control acid and base addition, while the DO signal drives feedback loops that adjust aeration rate, agitation speed, or backpressure to maintain a predefined setpoint [17].

For clarity, conventional monitoring tools in bioprocessing can be broadly grouped into three categories [20]. The first category comprises inline electrochemical probes, primarily glass pH electrodes and electrochemical DO sensors, that are permanently installed in the bioreactor and provide continuous measurements during the run. The second category consists of off-line and at-line analytical instruments, such as benchtop pH meters, blood-gas analyzers, and biochemical analyzers, which require manual sampling but can offer high accuracy and additional parameters that are not monitored online [21]. The third category includes single-use and miniaturized probe formats derived from traditional electrochemical designs, which are pre-mounted in disposable bags or integrated into small-volume cultivation platforms to support high-throughput process development [22,23]. Together, these three classes of tools constitute the conventional framework for pH and DO monitoring in current bioprocess practice. Table 2 summarizes the main features, advantages, and limitations of these categories in the context of bioreactor operation.

### 2.2. Limitations of Traditional Monitoring Methods

As highlighted in Table 2, each category of traditional monitoring tools exhibits characteristic strengths and weaknesses. However, several general limitations become particularly relevant when these methods are applied to modern and intensified bioprocesses, including susceptibility to environmental interference, a heavy calibration burden, and limited adaptability to non-conventional reactor formats.

#### 2.2.1. Environmental Interference

Although electrochemical pH and DO probes are well established, their performance can be strongly affected by the surrounding environment. Probe response depends on temperature and pressure, and while electronic compensation can correct part of the induced error, rapid or large changes in operating conditions may still lead to transient signal fluctuations or long-term drift [24]. The sensing surfaces are directly exposed to complex culture media that contain cells, proteins, metabolites, and gas bubbles; fouling, bubble adhesion, and changes in medium viscosity can all alter the effective mass transfer to the electrode or glass membrane [25]. As a result, the indicated values may deviate from the true conditions inside the bioreactor, especially in high–high-cell-density or long-duration processes, unless frequent maintenance and recalibration are performed [26].

#### 2.2.2. Regular Calibration Requirement

Reliable use of traditional pH and DO probes relies on strict calibration routines. Glass electrodes exhibit gradual changes in slope and offset due to aging of the glass membrane and alterations of the reference junction, so regular calibration with standard buffer solutions is required to maintain accuracy [27]. Electrochemical DO probes, in turn, require membrane inspection, electrolyte replacement, and polarization checks at defined intervals [28]. In regulated manufacturing environments, all these activities must be documented under good manufacturing practice (GMP), which increases labor and reactor downtime [19]. When many reactors are operated in parallel, or when small-scale high-throughput systems are employed for process development, this calibration workload can become a significant bottleneck and delayed, or incomplete calibration may compromise data quality [15].

#### 2.2.3. Adaptability Concerns

The adaptability of traditional monitoring methods is limited when bioprocessing moves beyond standard stainless-steel bioreactors. Conventional pH and DO probes are relatively bulky and are designed to fit standardized ports, which complicate integration into miniaturized or non-conventional culture systems such as microbioreactors, shake flasks, wave bags, or microfluidic devices [28]. Even in large production reactors, only a few probe positions are typically available, so inline electrochemical sensors provide essentially single-point measurements and cannot directly capture spatial gradients in pH or DO that arise from imperfect mixing or heterogeneous gas–liquid mass transfer [29]. Sampling-based off-line measurements partly compensate for these blind spots, but they are discontinuous, introduce time delays, and add risks of contamination and handling errors [30]. These constraints limit the usefulness of traditional methods in applications that require higher spatial resolution, dense sensor networks, or minimally invasive monitoring strategies [31,32].

In summary, conventional electrochemical probes and sampling-based analytical methods remain the workhorse for pH and DO monitoring in bioprocessing, but they suffer from well-known limitations. Drift, fouling, frequent calibration, and the need for manual sampling restrict their suitability for intensive monitoring and scale-down studies. In addition, bulky probe geometries, limited port availability, and sterility constraints make it difficult to implement these tools in small-scale, single-use, or non-conventional bioreactor formats. These shortcomings motivate the development of compact, minimally invasive optical fiber–based sensing solutions for future bioreactor applications.

## 3. Overview of Optical Fiber Sensor Technology

### 3.1. Basic Principles of Optical Fiber Sensors

Optical fiber sensors convert changes in the surrounding physical, chemical, or biological environment into variations in light guided in an optical fiber [33]. A standard optical fiber consists of a high-index core, a lower-index cladding, and a protective coating. Light is confined in the core by total internal reflection, and the measurand can be encoded as changes in intensity, phase, polarization, or wavelength of the guided modes [33,34,35]. For bioprocess monitoring, the sensing region is usually created by modifying the fiber structure (e.g., tapering, etching, creating microcavities, or inscribing gratings) and/or by depositing functional coatings that respond to analytes such as pH or DO [35,36,37,38,39]. 

**Figure 1:**
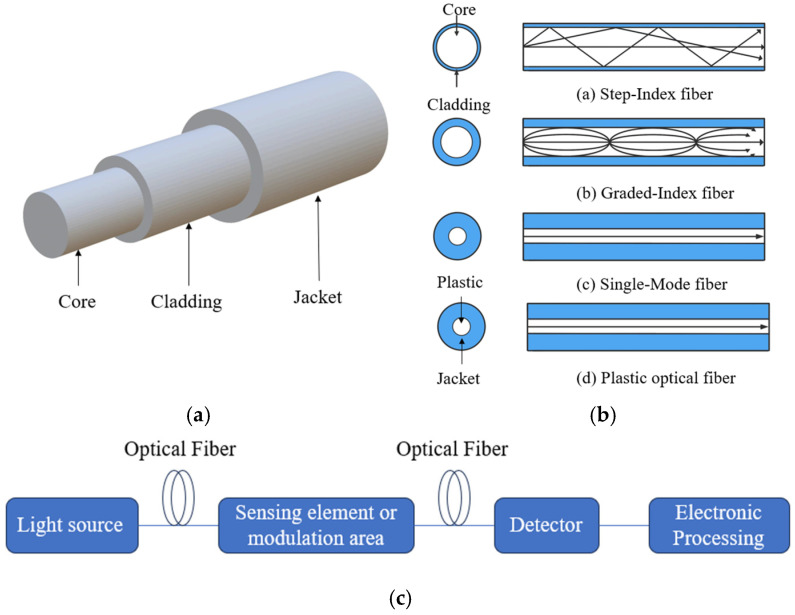
The basic structure of optical fibers. (**a**) Structure of optical fiber; (**b**) Different types of fibers; (**c**) Schematic of an optical fiber sensor system.

Figure 1 schematically illustrates the basic structure of a standard step-index optical fiber, examples of different fiber types, and the main components of an optical fiber sensor system. Beyond conventional single-mode and multimode silica fibers, a variety of specialty fibers have been developed, including tapered microfibers, polymer optical fibers, microstructured or photonic crystal fibers, and hollow-core anti-resonant fibers [36]. Tapered fibers and microfibers enhance the evanescent field and light–matter interaction, which can significantly improve sensitivity to refractive index or coating-induced changes [37]. Microstructured and hollow-core fibers offer flexible guidance mechanisms and long interaction lengths with gases or liquids and have been widely investigated in optofluidic and biochemical sensing [38]. However, these specialty fibers are typically more expensive and fragile than standard telecom-grade fibers and can be more difficult to fabricate, splice, and handle, which limits their widespread use in routine bioprocess monitoring [39]. As a result, many optical fiber pH and DO sensors for bioreactor applications still rely on single-mode or multimode silica fibers or robust plastic optical fibers as a platform [40].

From the viewpoint of transduction mechanisms, optical fiber sensors can be broadly classified as intensity-based, wavelength-encoded, phase-based, and polarization-based devices [41]. Intensity-based sensors track changes in transmitted, reflected, or fluorescence intensity, often by immobilizing indicator dyes near the fiber surface or within a coating. Wavelength-encoded sensors rely on spectral shifts of interference fringes, Fabry–Pérot resonances, long-period fiber gratings, fiber Bragg gratings, or surface plasmon resonances [42]. Phase-based and polarization-based configurations detect measurand-induced changes in the phase difference or polarization state of guided modes, for example, in Mach–Zehnder, Sagnac, or polarization-maintaining fiber interferometers [43,44]. Table 3 summarizes the main sensing mechanisms, typical fiber structures, encoded optical quantities, and their advantages and limitations in the context of pH, DO, and related bioprocess parameters [33,34,35,36,37,38,39,40,41,42,43,44].

### 3.2. Advantages of Optical Fiber Sensors

#### 3.2.1. High Sensitivity

Optical fiber sensors can achieve high sensitivity because light is tightly confined in the fiber over long interaction lengths, and small perturbations in the surrounding medium or in functional coatings can induce measurable changes in the guided modes [33,34,35,36]. Interferometric configurations, grating-based structures (such as FBGs and LPFGs), plasmonic fiber probes based on surface plasmon resonance (SPR), and graphene-enhanced devices have all demonstrated high sensitivity to refractive index, absorption, and surface binding events in a variety of chemical and biochemical sensing applications [42,43]. For pH and DO monitoring, the combination of responsive indicator materials with evanescent-field, interferometric, or grating-based transduction enables the detection of relatively small deviations from the target operating window, which is important for maintaining optimal cell growth and product quality in bioprocesses [44]. In addition, the use of advanced coating materials and nanostructured interfaces can further enhance the responsivity and limit of detection of optical fiber sensors without substantially increasing their physical footprint [44,45,46].

#### 3.2.2. Remote Sensing Capability

Because the sensing signal is carried optically, optical fiber sensors are inherently suited for remote measurements, where the interrogation unit can be physically separated from the sensing region by distances ranging from a few meters to many kilometers [47]. Only the fiber and sensing elements need to be in direct contact with the bioreactor environment, while the light source and detection electronics remain in a benign location. This separation reduces the exposure of electronic components to high temperature, pressure, humidity, or corrosive chemicals and facilitates integration with closed or pressurized vessels [47,48,49]. Furthermore, optical fibers support distributed and quasi-distributed sensing approaches based on Rayleigh, Brillouin, or Raman scattering, as well as multiplexed grating arrays, which enable spatially resolved monitoring along the entire fiber length [48,49]. Such capabilities are attractive for future bioprocess applications that may require multi-point temperature profiling, detection of gradients, or simultaneous measurement of several critical parameters within large-scale reactors or across connected unit operations.

#### 3.2.3. Miniaturization and Flexibility

Standard silica and polymer optical fibers typically have outer diameters on the order of 100–250 μm and can be fabricated into flexible, bend-tolerant probes, enabling deployment in confined or complex geometries [50]. Their small size and mechanical flexibility allow insertion through narrow ports, embedding within sampling lines or mixing elements, and integration into microfluidic or optofluidic platforms with minimal disturbance of flow patterns and working volume [51]. Plastic optical fibers, in particular, offer low-cost, mechanically robust, and easily cuttable options that are attractive for disposable probes and small-scale or single-use systems [47,50]. Beyond bioprocessing, optical fiber sensors have been successfully applied in environmental and water quality monitoring, marine sensing, and structural health monitoring, demonstrating long-term robustness and operation under harsh conditions [47,48]. These attributes—miniaturization, flexibility, compatibility with disposable and small-volume systems, and proven robustness in demanding environments—make optical fiber sensors strong candidates to complement or replace conventional electrochemical pH and DO probes and to support future multi-parameter sensing concepts within PAT and QbD frameworks [51].

## 4. Application of Optical Fiber pH Sensors in Bioreactors

### 4.1. Significance of pH in Bioprocessing Processes

In both microbial fermentation and mammalian cell culture, pH is widely recognized as one of the most critical process parameters because it directly influences intracellular enzyme activity, membrane transport, nutrient uptake, and overall cell viability [52,53]. For mammalian systems such as CHO cell cultures used in monoclonal antibody production, productive operation is typically confined to a relatively narrow pH window (≈6.8–7.4); even small deviations can alter cell growth kinetics, specific productivity, and product quality attributes such as glycosylation profiles [53,54]. Consequently, most industrial bioreactors are equipped with automated pH control loops that continuously adjust acid/base or CO_2_ stripping to maintain the culture within a predefined range [53,55].

Beyond its impact on cell growth, pH also modulates central carbon and nitrogen metabolism. Numerous studies have shown that culture pH and its temporal profile are tightly coupled to lactate and ammonia accumulation, which in turn affect energy metabolism, osmolarity, and antibody productivity [52,56]. For example, elevated ammonia and lactate concentrations have been reported to inhibit cell growth, modify intracellular pH, and change metabolic flux distributions, while dynamic pH-control strategies can be used to steer cells toward more favorable metabolic states with reduced by-product formation [56]. Optimized pH-shift or pH-profile strategies have been successfully applied to increase CHO cell titers while maintaining or improving critical quality attributes (CQAs) such as N-glycan distribution [53,54,55].

From a QbD and PAT perspective, pH is therefore routinely classified as a critical process parameter (CPP) because its fluctuations propagate to multiple CQAs, including glycosylation, charge variants, and impurity profiles [57]. Modern PAT frameworks emphasize real-time measurement and control of CPPs such as pH, DO, and key metabolite concentrations to maintain the process within a well-defined design space and to support continuous process verification [58]. However, conventional pH sensors typically provide only single-point information and may be difficult to integrate into emerging miniaturized or single-use cultivation platforms. This creates a strong motivation to explore alternative technologies—such as optical fiber sensors—that can offer a small physical footprint, enhanced robustness, and multiplexed or distributed pH monitoring capabilities to better support advanced PAT-enabled bioprocessing [58].

### 4.2. Working Principle of Optical Fiber pH Sensors

Optical fiber pH sensors convert changes in hydrogen ion activity into measurable variations in light guided within or around the fiber. In general, this is achieved by combining a pH-responsive material with an appropriate fiber structure so that local changes in protonation state modify the absorption, fluorescence, refractive index, or effective optical path length of the sensing region [59,60,61,62]. Depending on the transduction scheme, optical fiber pH sensors can be broadly divided into three main classes: (i) indicator-based absorption or fluorescence sensors, (ii) hydrogel-based sensors that exploit pH-induced swelling and refractive-index changes, and (iii) interferometric and grating-based fiber sensors that convert these material changes into spectral shifts or phase variations. Representative implementations of these mechanisms include the early indicator-based systems reported by Holobar, Kermis, and Weidgans [59], and the more recent hydrogel- and interferometer-based designs developed by Gerlach, Gu, Corres, Richter, and Pathak [63,64,65,66,67,68,69].

#### 4.2.1. Indicator-Based Absorption and Fluorescence Sensors

The most established approach to fiber-optic pH sensing employs pH-sensitive dyes or fluorescent indicators immobilized on or near the fiber surface. Many organic indicators exist in at least two prototropic forms (protonated and deprotonated) with distinct absorption and emission spectra. Their relative populations are governed by the Henderson–Hasselbalch relationship, so the absorbance or fluorescence signal follows a sigmoidal dependence on pH centered around the indicator pKa. In intensity-based fiber sensors, the fiber delivers excitation light to the indicator layer and collects the reflected or emitted light; changes in absorbance or fluorescence intensity with pH are then correlated with the local pH via calibration [39].

Early work by Holobar et al. and subsequent studies by Kermis and Weidgans demonstrated that such indicator-based designs can be implemented in fiber geometries suitable for non-invasive or minimally invasive measurements in bioreactors and small-scale culture systems [59,60,61,62]. However, the basic transduction principle remains the same: the optical signal reflects the acid–base equilibrium of the immobilized indicator at the sensor surface. To mitigate the influence of fluctuations in source power, coupling efficiency, and fiber bending losses, many designs use ratiometric schemes comparing a pH-sensitive band with a reference band, or employ dual-excitation/dual-emission configurations [59,60,61,62]. In addition, fluorescence lifetime-based readout can be used instead of absolute intensity, since lifetime is largely independent of optical losses in the fiber and therefore less sensitive to fouling or alignment changes. In all cases, careful immobilization of the indicator in matrices such as sol–gel, polymers, or hydrogels is required to prevent dye leaching and to ensure fast and reversible response in aqueous culture media [59,60,61,62].

#### 4.2.2. Hydrogel-Based Swelling and Refractive-Index Sensors

Hydrogel-based pH sensors use crosslinked polymer networks containing ionizable groups whose degree of dissociation depends on pH [63]. When these groups become charged, electrostatic repulsion and osmotic pressure changes drive water uptake, causing the hydrogel to swell; when the groups are neutralized or screened, the network can shrink. These pH-induced volume transitions modulate both the physical thickness and the effective refractive index of the hydrogel layer, which in turn affects how light propagates in adjacent optical structures [68].

In fiber-based implementations, hydrogels can be coated on etched or tapered fiber segments, deposited over interferometric cavities, or integrated with surface-relief and photonic structures [63,64,65]. Swelling or shrinking of the coating changes the evanescent-field overlap, the index contrast between core, cladding, and surrounding medium, or the cavity length, giving rise to measurable variations in transmitted intensity, reflected power, or resonance wavelength [63,64,65,66,67,68,69]. Gerlach and co-workers systematically analyzed hydrogel swelling as a basis for pH sensing, while subsequent work by Shin and others used structured hydrogel layers such as inverse opal films to enhance the optical response [63,65]. Pathak and Singh implemented a polyacrylamide hydrogel coating on a no-core fiber interferometer, illustrating how volumetric changes in the hydrogel can be transduced into wavelength shifts in compact fiber structures [69]. Overall, hydrogel-based platforms provide a versatile route to pH sensing in water-rich and biologically relevant environments and can be tailored to specific pH ranges and response characteristics through appropriate selection of monomers, crosslinkers, and functional groups.

#### 4.2.3. Interferometric and Grating-Based Fiber Sensors

Interferometric and grating-based designs provide another powerful route for optical fiber pH sensing by converting pH-dependent refractive-index or thickness changes into spectral shifts or phase variations. In modal interferometers—such as no-core fiber or thin-core fiber interferometers—a section of specialty fiber is spliced between two single-mode fibers. At the first splice, multiple core and cladding modes are excited; these modes accumulate different phase delays along the sensing section and interfere at the second splice. When a pH-sensitive coating is applied to the cladding region, changes in its refractive index or thickness alter the effective indices and confinement of the cladding modes, thereby shifting the interference fringes in wavelength or phase [64,69]. Gu and co-workers used a thin-core fiber interferometer with an electrostatically assembled polyelectrolyte nanocoating as a representative example of this approach [64].

Long-period fiber gratings (LPFGs) and related grating structures operate on a similar principle. An LPFG couples light from the core mode into co-propagating cladding modes at specific resonance wavelengths determined by the phase-matching condition between the modes and the grating period. Coating the grating region with a pH-responsive overlay changes the surrounding refractive index and the effective indices of the cladding modes, leading to measurable shifts in the attenuation bands [66,67]. Elster and Corres demonstrated that electrostatically self-assembled polyelectrolyte coatings on LPFGs can be used to realize pH-sensitive gratings, and that coupled-mode analysis provides a framework to relate the spectral response to overlay properties and pH [66,67]. Similar principles have been applied in other interferometric geometries, where pH-responsive layers modulate the optical path length or reflectivity of the cavity, yielding wavelength- or phase-encoded pH readouts in compact fiber-based probes [63,64,65,66,67,68,69].

In summary, optical fiber pH sensors rely on well-understood photophysical and physicochemical mechanisms—indicator protonation, hydrogel swelling, and pH-dependent refractive-index changes—implemented within fiber-compatible structures such as coated straight fibers, interferometers, and gratings.

### 4.3. Advantages of Optical Fiber pH Sensors

As outlined in Section 2, conventional electrochemical pH probes face limitations in terms of calibration burden, contamination risk, and compatibility with miniaturized or single-use bioreactors. Optical fiber pH sensors address many of these issues through non-invasive, all-optical operation and highly adaptable probe geometries, as summarized in the following subsections.

#### 4.3.1. Non-Invasive Measurement

Optical fiber pH sensors operate purely on optical signals and do not require bulky electrodes or direct electrical contact with the culture medium. The sensing element can be configured as a small coated region on the fiber surface, a miniaturized flow-through cell, or an external patch interrogated via an optical fiber [33,34,35,36,39]. Because only light is transmitted through the fiber, the measurement can be performed at a distance from the culture vessel, while the sensing area itself can be made very small and mechanically unobtrusive. Early fiber-based pH systems, such as the flow-through configuration reported by Holobar et al., already demonstrated that optical interrogation can track pH changes without introducing significant dead volume or mechanical disturbance to the culture [59]. These features are particularly advantageous in small-scale reactors, microbioreactors, and microfluidic devices where invasive probes would perturb hydrodynamics or consume a non-negligible fraction of the working volume [59,60,61,62].

#### 4.3.2. No Contamination Risk

Because optical fiber sensors do not rely on liquid-filled reference junctions or permeable membranes, they eliminate several contamination pathways associated with traditional glass electrodes and electrochemical probes [52,53,54,55,56,57,58]. The sensing region typically consists of a solid-state indicator or hydrogel coating immobilized on the fiber and can be permanently sealed within the reactor or integrated into pre-sterilized disposable components [63,64,65,66,67,68,69]. No electrolyte reservoir can leak into the culture, and the absence of metallic electrodes reduces the risk of corrosion products or leachables. In addition, the small size of the fiber allows the design of closed, weldable connections in single-use systems, which helps maintain sterility throughout long-term cultivations. These characteristics make optical fiber pH sensors well-suited for applications that demand strict contamination control, including mammalian cell culture for therapeutic protein production and organ-on-chip platforms.

#### 4.3.3. Rapid Response Time

The small dimensions of the sensing layer and the high surface-to-volume ratio of the fiber geometry contribute to fast mass transfer of protons between the bulk medium and the indicator or hydrogel, enabling rapid response times [59,60,61,62,63,69]. Fluorescent indicator-based designs can track pH fluctuations on the order of seconds or faster, which is sufficient to capture typical process dynamics in stirred-tank and perfusion bioreactors [62]. Interferometric and grating-based sensors likewise translate small and rapid changes in refractive index or coating thickness into measurable spectral shifts with high temporal resolution [64,65,66,67]. Compared with off-line or at-line measurements, which inherently introduce time delays due to sampling and analysis, optical fiber pH sensors can therefore provide near-continuous profiles of pH evolution. This enables tighter feedback control, improved detection of transient events such as feeding-induced excursions, and more accurate characterization of process dynamics for model-based control and QbD studies [52,53,54,55,56,57,58].

#### 4.3.4. Reduced Maintenance

Traditional pH electrodes suffer from gradual drift in slope and offset, as well as fouling and aging of the reference junction, which necessitate frequent calibration and maintenance to ensure reliable operation [52,53,54,55,56,57,58]. Optical fiber pH sensors replace the fragile glass membrane and liquid reference system with robust solid-state coatings, reducing the number of consumable parts and eliminating electrolyte handling [59,60,61,62]. Although indicator photobleaching, coating degradation, and hydrogel aging can still lead to signal drift, these processes are typically slower and more predictable, and many designs have demonstrated stable operation over multiple batches or extended cultivation periods [59,60,61,62,63,68,69]. In addition, fluorescence lifetime and ratiometric interrogation schemes further mitigate the impact of optical losses, fouling, and source fluctuations, decreasing the frequency and complexity of recalibration. Overall, this translates into lower maintenance burden and reduced downtime, especially when numerous reactors are operated in parallel.

#### 4.3.5. Adaptability to High-Density Cultivation

In high–high-cell-density processes, gradients of pH and dissolved CO_2_ can develop due to incomplete mixing, gas–liquid mass-transfer limitations, or localized metabolic activity. Conventional single-point pH probes provide only limited insight into these spatial heterogeneities [52,53,54,55,56,57,58]. By contrast, optical fiber pH sensors can be deployed at multiple positions within the same vessel, along baffles or spargers, or in different zones of a scaled-down model, enabling more detailed mapping of pH distributions [64,65,66,67,68,69]. Interferometric and grating-based designs can also be combined with distributed temperature and strain sensing along the same fiber, providing additional context for interpreting pH fluctuations in heterogeneous environments [47,48,49]. Such capabilities are valuable for understanding scale-up effects, validating computational fluid dynamics (CFD) models, and designing control strategies that are robust to local gradients. Moreover, hydrogel-based sensors retain functionality in complex media containing cells, proteins, and metabolites, making them suitable for long-term monitoring in dense mammalian or microbial cultures [68,69].

#### 4.3.6. Flexible Deployment

The intrinsic flexibility and small diameter of optical fibers facilitate their integration into a wide variety of bioprocess formats, ranging from milliliter-scale microbioreactors and microfluidic chips to bench-top glass bioreactors and large stainless steel or single-use production systems [62]. Fibers can be routed through standard probe ports, welded connectors, or custom-designed holders, and can be configured as probe-type sensors, wall-mounted patches, or flow-through cells, as illustrated in the pH sensing assemblies discussed in the next subsection. Furthermore, the same fiber platform can often be adapted to host multiple sensing chemistries—such as pH, DO, and specific analytes—on different segments or within different optical structures (e.g., gratings and interferometers), enabling compact multi-parameter probes [64,65,66,67,68,69]. This flexibility supports the development of integrated monitoring solutions that address several critical process parameters simultaneously and facilitate the transition from laboratory-scale experiments to industrial implementation.

In summary, optical fiber pH sensors offer a combination of non-invasive operation, low contamination risk, rapid response, reduced maintenance, adaptability to high-density cultures, and flexible deployment that is difficult to achieve with conventional electrochemical probes. These advantages, together with the diverse transduction mechanisms outlined in Section 4.2 and the concrete implementations reviewed in Section 4.4, highlight the strong potential of optical fiber pH sensing as a core component of future PAT-enabled bioprocess monitoring strategies [52,53,54,55,56,57,58,59,60,61,62,63,64,65,66,67,68,69].

### 4.4. Typical Application Cases of Optical Fiber pH Sensors

#### 4.4.1. Indicator-Based Fiber pH Sensors in Bioreactors and Shake Flasks

**Figure 2:**
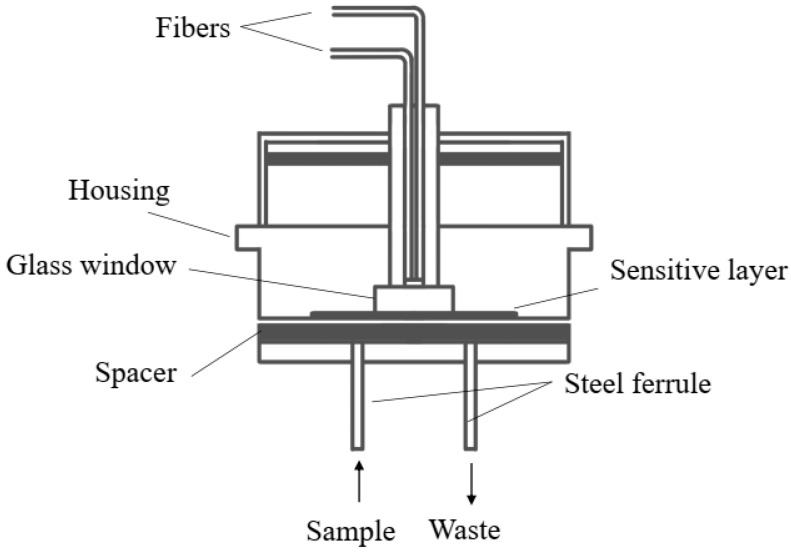
Schematic cross-section of the flow-through unit employed for pH sensor characterization.

**Figure 3:**
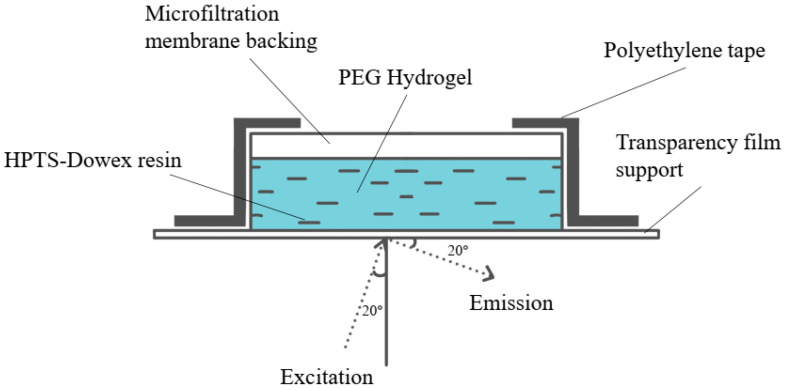
Cross-sectional view of the flow-through cell used for pH sensor evaluation.

Indicator-based fluorescence and absorption designs are the most mature class of optical fiber pH sensors for bioprocessing. They exploit the protonation-dependent spectra of immobilized dyes, as described in Section 4.2.1, and have been implemented in several geometries that minimize intrusiveness while maintaining adequate mass transfer [70,71,72,73,74,75]. A typical approach is to confine the pH-sensitive layer in a small flow-through unit or flow cell that is inserted into a bypass loop connected to the main bioreactor. Figure 2 shows the cross-section of such a flow-through unit used for pH sensor characterization [48], while Figure 3 illustrates a related flow-through cell design [59]. In both cases, the culture broth is continuously circulated past the optical sensing region, allowing near real-time monitoring without placing bulky probes directly inside the vessel.

Holobar et al. demonstrated one of the earliest optical fiber pH measurement systems for bioreactors using a fluorescent indicator coated in a flow-through configuration coupled to a bench-top reactor [59]. Subsequent work by Kermis and co-workers refined this concept by introducing dual-excitation ratiometric readout and robust immobilization procedures, thereby improving signal stability and reducing sensitivity to fluctuations in source intensity and fiber alignment [49,50]. Weidgans et al. further addressed ionic-strength effects by developing fluorescent indicators with negligible sensitivity to electrolyte composition, which is important for media formulations with varying salt content [51]. Shen et al. reported a long-wavelength fluorescent hydrophilic copolymer with a broad linear response range, facilitating accurate pH monitoring over extended windows [54], while Janzen et al. evaluated fluorimetric pH sensors for challenging low-pH fermentation processes [55]. Mousavi Shaegh et al. integrated optical pH and oxygen sensing into a microfluidic platform for real-time monitoring of microfluidic bioreactors and organ-on-chip devices [56], and Newton et al. developed process-adapted calibration strategies to improve the accuracy of fluorometric pH sensors in complex fermentation environments [57]. Demuth et al. and Udomsom et al. further illustrated how novel optical probes and automatic programmable systems can support pH monitoring across scales and in tissue engineering applications [52,53]. Overall, these studies show that indicator-based fiber and fiber-coupled sensors can reach accuracies comparable to glass electrodes while offering non-invasive or minimally invasive operation, straightforward optical readout, and good compatibility with sterilizable flow components. Representative implementations, including sensing mechanisms, bioreactor formats (shake flasks, microreactors, and stirred-tank bioreactors), and key qualitative outcomes of these optical pH sensors are summarized in Table 4.

**Figure 4:**
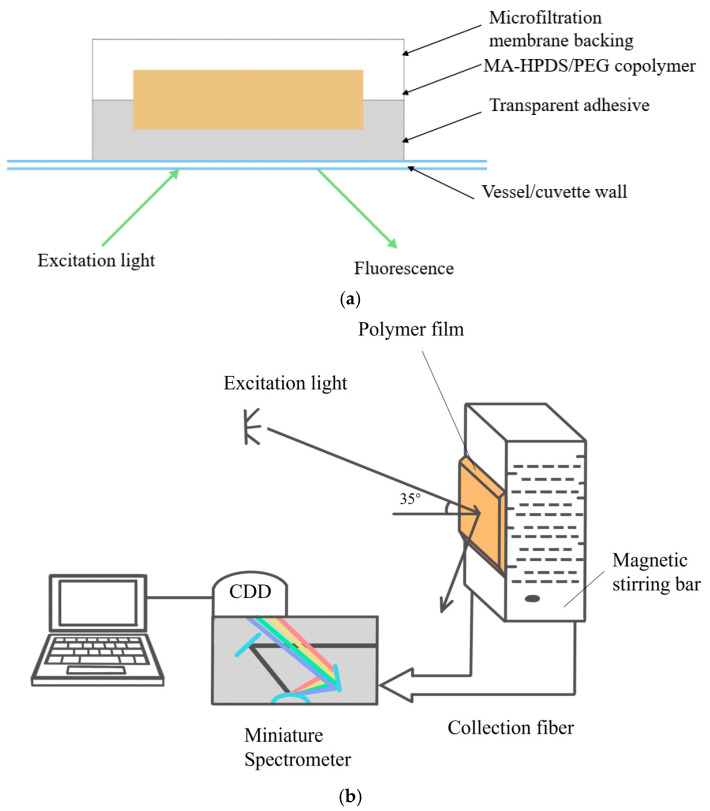
(**a**) Schematic illustration of the HPTS-PEG-Dowex sensing structure mounted on a transparency substrate. (**b**) Diagram of the fluorescence-based pH measurement setup, showing the cuvette with front-face excitation and emission detection for online pH determination.

In addition to flow-through geometries, indicator-based fiber pH sensors have been integrated into disposable or modular sensing assemblies suitable for small-scale and high-throughput cultivation. Figure 4a depicts an example of an HPTS-PEG-Dowex sensing head that combines a pH-sensitive fluorophore with an ion-exchange matrix and polymer support [59], while Figure 4b shows the corresponding optical arrangement for excitation and emission collection [54]. Such assemblies can be incorporated into miniaturized bioreactors, microtiter plates, or disposable flow cells, providing flexible sensor formats for screening studies and process development. Overall, indicator-based optical fiber pH sensors offer a relatively low barrier to implementation because they are conceptually close to traditional optical pH probes, yet benefit from the small footprint and remote readout capabilities of fibers.

#### 4.4.2. Interferometric and Grating-Based Fiber pH Sensors

Interferometric and grating-based optical fiber pH sensors convert pH-induced changes in coating refractive index or thickness into spectral shifts or phase variations, as outlined in Section 4.2.3. Compared with purely intensity-based designs, these architectures often provide higher spectral resolution and can be more robust to intensity fluctuations, making them attractive for applications where long fiber leads, multi-point sensing, or harsh environmental conditions are involved.

**Figure 5:**
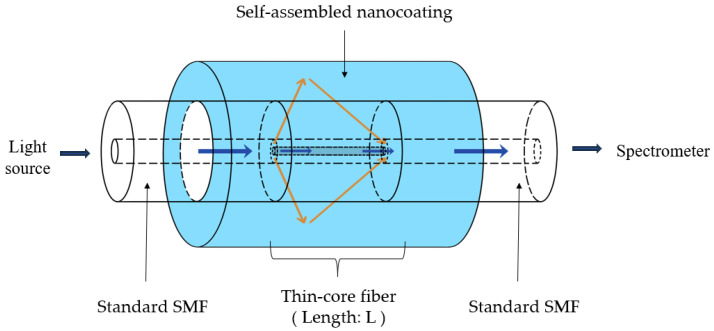
Structural illustration of the thin-core fiber interferometer–based pH sensor incorporating an electrostatically assembled nanocoating.

Figure 5 schematically illustrates a thin-core fiber interferometric pH sensor incorporating an electrostatically assembled nanocoating on the cladding region. In such sensors, a section of thin-core fiber is spliced between two single-mode fibers, exciting multiple modes that accumulate differential phase delays along the sensing region [65]. When a pH-responsive polyelectrolyte overlay is deposited on the cladding, its protonation-dependent refractive index and thickness alter the effective indices of the cladding modes, causing measurable wavelength shifts of the interference fringes. Gu and co-workers used this configuration as a compact fiber probe, demonstrating that pH-induced spectral shifts can be interrogated with simple broadband sources and spectrometers [64]. Similar interferometric designs can be embedded in small flow cells, inserted through standard probe ports, or integrated into sampling lines, providing flexible options for in-line pH monitoring in complex bioprocess setups.

Long-period fiber gratings (LPFGs) offer another route for grating-based pH sensing. In LPFGs, the core mode is coupled to co-propagating cladding modes at discrete resonance wavelengths defined by the grating period and the effective indices of the modes. Coating the grating region with a pH-responsive overlay changes the surrounding refractive index and therefore shifts the resonance wavelengths [66,67]. Elster and Corres functionalized LPFGs with electrostatically self-assembled polyelectrolyte multilayers and used coupled-mode theory to link the observed spectral changes to overlay properties and pH. Although many LPFG-based pH sensors have been demonstrated in buffered solutions or simple media, their underlying principles are directly applicable to bioreactor environments, especially when compact probes or multiplexed multi-point measurements are desired.

#### 4.4.3. Hydrogel-Based Fiber pH Sensors

Hydrogel-based designs exploit pH-dependent swelling and refractive-index changes in crosslinked polymer networks, which can be transduced into optical signals by appropriate fiber configurations, as discussed in Section 4.2.2. The hydrogel typically contains weakly acidic or basic groups whose ionization state modulates the osmotic pressure and electrostatic interactions within the network. As pH changes, the hydrogel swells or shrinks, altering its thickness, water content, and refractive index; these physical changes can be sensed through evanescent-field interactions or mechanical coupling to the fiber.

**Figure 6:**
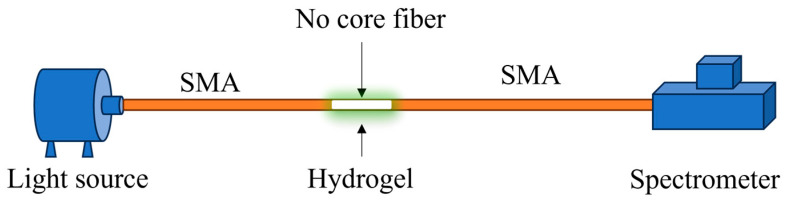
Diagram of the constructed sensing probe featuring a hydrogel-coated detection region.

Figure 6 shows a representative sensing probe design featuring a hydrogel-coated detection region [69]. In many implementations, the fiber is tapered, etched, or microstructured to enhance evanescent-field penetration into the surrounding hydrogel layer, thereby increasing sensitivity to changes in its optical properties [63,68,69]. Gerlach and colleagues systematically investigated hydrogel swelling as a basis for chemical and pH sensing, providing design guidelines for selecting polymer compositions and crosslink densities [63]. Shin and co-workers employed inverse opal hydrogel structures with tunable photonic band gaps, in which pH-induced volume changes produced colorimetric or spectral responses [65]. Pathak and Singh combined a polyacrylamide hydrogel coating with a no-core fiber interferometer, demonstrating wide-range pH sensing and good repeatability in an all-fiber format [69]. Richter and co-workers reviewed a broad spectrum of hydrogel-based pH sensors and microsensors, highlighting strategies for improving response time, stability, and biocompatibility [68]. Such hydrogel-based fiber sensors are particularly attractive for biological and microfluidic applications because they are inherently water-compatible and can be engineered for specific pH windows and response speeds. Their compatibility with soft materials and small geometries makes them promising candidates for integration into organ-on-chip devices, perfusion microbioreactors, and other emerging culture platforms.

#### 4.4.4. Comparative Assessment and Design Considerations

The application cases reviewed above highlight that optical fiber pH sensors can be tailored to very different bioprocess scenarios by combining indicator-based coatings, interferometric or grating structures, and hydrogel architectures [70,71,72,73,74,75,76]. Indicator-based designs are attractive for straightforward implementation and intuitive calibration but may suffer from photobleaching and limited long-term stability. Interferometric and grating-based sensors offer high sensitivity and compact footprints, yet they often require more elaborate optical interrogation. Hydrogel-based probes are particularly promising for miniaturized and perfusion systems because they allow highly localized sensing in small volumes. From a design perspective, selecting an appropriate architecture requires balancing the target pH range and resolution, compatibility with the bioreactor format, optical hardware complexity, and the expected lifetime and robustness of the sensing layer.

## 5. Application of Optical Fiber DO Sensors in Bioreactors

### 5.1. Significance of DO in Bioprocessing Processes

DO is a critical process parameter in aerobic bioprocesses because it directly affects cellular respiration, energy generation, biomass formation, and product quality. In microbial fermentations and mammalian cell cultures, oxygen serves as the terminal electron acceptor in oxidative metabolism; insufficient DO rapidly leads to reduced ATP supply, shifts toward anaerobic or overflow metabolism, and accumulation of by-products, whereas excessive DO can promote oxidative stress and damage to sensitive biomolecules [76,77]. As summarized in Table 1, different organism types exhibit distinct optimal DO ranges and oxygen uptake characteristics, so DO control strategies must be tailored to the specific host and cultivation mode [78].

In large-scale stirred-tank bioreactors, maintaining homogeneous DO is challenging due to limitations in gas–liquid mass transfer, mixing, and local hydrodynamics. Scale-up often amplifies DO gradients between well-aerated regions and poorly mixed zones, which can expose cells to fluctuating oxygen levels and cause variability in growth, metabolism, and critical quality attributes [77]. Miniaturized and high-throughput cultivation systems, such as parallel microbioreactors and microfluidic devices, also require precise DO control because small working volumes and high surface-to-volume ratios can accelerate oxygen depletion or oversupply [76,77,78]. Consequently, DO setpoints and control strategies are now routinely included in QbD studies and risk assessments for biopharmaceutical processes, alongside other critical parameters such as pH, temperature, and nutrient concentrations [79].

Traditional electrochemical DO probes remain the workhorse for DO control; however, the increasing use of single-use bioreactors, perfusion systems, and integrated continuous processes has highlighted their limitations in terms of sterilization, footprint, maintenance, and suitability for small-scale or complex geometries [79,80,81,82,83]. This has stimulated significant interest in optical DO sensing technologies that exploit oxygen-dependent luminescence quenching of immobilized dyes. Several families of oxygen-sensitive luminophores, including platinum porphyrins (e.g., PtOEP, PtTFPP) and ruthenium complexes (e.g., Ru(dpp)_3_^2+^, Ru(bpy)_3_^2+^), have been developed together with sol–gel, polymer, and composite immobilization matrices to tune sensitivity, response time, and operational stability [84,85,86,87,88,89,90,91]. An overview of typical immobilization materials, response characteristics, and sensitivity ranges reported for representative optical DO sensors is provided in the DO sensor performance table. These optical sensing chemistries form the foundation for the optical fiber DO sensors discussed in the following subsections, which aim to deliver robust, real-time DO monitoring across a wide variety of bioreactor formats. An overview of typical immobilization materials, response characteristics, and sensitivity ranges reported for representative optical DO sensors is provided in Table 5 [84,85,86,87,88,89,90,91].

### 5.2. Working Principles of Optical Fiber Dissolved Oxygen Sensors

Optical fiber dissolved oxygen sensors are predominantly based on dynamic luminescence quenching of oxygen-sensitive dyes immobilized in gas-permeable matrices and interrogated via optical fibers [33,34,35,36,39,40,41,84,85,86,87,88,89,90,91]. Compared with electrochemical probes, these sensors do not consume oxygen during measurement, can be miniaturized into thin films or small sensing spots, and are well-suited for remote, non-invasive monitoring in bioreactors and microbioreactors [76,77,78,79,80,81,82,83]. This subsection briefly summarizes the underlying quenching mechanism and the role of immobilization matrices, which together determine the sensitivity, response time, and operational robustness of optical fiber DO sensors.

#### 5.2.1. Oxygen-Dependent Luminescence Quenching

Most optical DO sensors employ phosphorescent or fluorescent transition-metal complexes whose excited states are efficiently quenched by molecular oxygen. Typical luminophores include platinum(II) porphyrins such as PtOEP and PtTFPP, as well as ruthenium(II) polypyridyl complexes such as Ru(dpp)_3_^2+^ and Ru(bpy)_3_^2+^ [84,85,86,87,88,89]. Upon excitation, these dyes exhibit emission with an intensity and/or lifetime that decreases as the local oxygen partial pressure increases due to collisional (dynamic) quenching. The relationship between the unquenched and quenched signals is commonly described by the Stern–Volmer equation, which links the ratio of emission intensity or lifetime to the DO concentration and an effective quenching constant [84,85,86,87,88]. At low to moderate oxygen levels and in homogeneous environments, this relationship is approximately linear, enabling straightforward calibration. Deviations from linearity are often observed at high oxygen concentrations or in heterogeneous matrices and can be captured by modified Stern–Volmer models or multi-site approaches [90,91].

For fiber-coupled sensors, the excitation light is delivered through a multimode or single-mode optical fiber to the sensing region, where it is absorbed by the immobilized dye. The resulting emission is partly coupled back into the same or a separate collection fiber and guided to a photodetector or spectrometer [84,85,86,87,88,89,90,91]. Depending on the interrogation scheme, the sensor can operate in intensity mode, lifetime mode, or phase-modulation mode. Intensity-based readout monitors changes in the steady-state emission amplitude, whereas lifetime and phase-modulation techniques measure changes in the excited-state decay time or the phase shift of a modulated signal, which are less sensitive to fluctuations in light source intensity, fiber bending, or fouling of optical surfaces [84,85,86,87,88]. Ratiometric and referencing strategies, for example, using dual-wavelength excitation or co-immobilized reference dyes, further improve robustness by compensating for non-analyte-related signal variations [90,91].

#### 5.2.2. Immobilization Matrices and Fiber Integration

The performance of optical fiber DO sensors is strongly influenced by the choice of immobilization matrix that hosts the luminophore. The matrix must provide sufficient oxygen permeability to enable rapid diffusion, protect the dye from leaching and chemical degradation, and maintain mechanical integrity and adhesion to the fiber or substrate under bioreactor conditions [84,85,86,87,88,89]. Early optical DO sensors incorporated PtOEP in elastic fluorinated polymers to achieve high oxygen solubility and fast response times, while subsequent work explored sol–sol–gel-derived silica, organically modified silicates (ormosils), xerogels, and composite coatings to tune sensitivity, dynamic range, and photostability [84,85,86,87,88,89]. These matrices can be formulated to cover different DO ranges, from low-oxygen microenvironments to air-saturated or oxygen-enriched media, by adjusting polymer polarity, crosslink density, and dye loading [84,85,86,87,88,89,90,91]. 

In fiber-based implementations, the oxygen-sensitive layer can be integrated in several geometries. One common configuration uses an extrinsic sensor tip, where the dye-doped film is deposited on the end face or side-polished region of a multimode fiber; the fiber serves purely as a light guide, while the sensing chemistry resides in a separate coating or cap. Alternatively, planar sensor patches incorporating the luminophore can be attached to the inner or outer wall of a bioreactor or shake flask, with optical fibers or lens–fiber assemblies placed at a distance for excitation and collection, enabling non-invasive measurements through transparent vessel walls. Such arrangements are particularly attractive for single-use bags, microbioreactors, and microfluidic devices, where avoiding direct penetration of the culture compartment is desirable [76,77,78,79,80,81,82,83]. In all cases, the combination of oxygen-diffusible matrices, well-characterized luminescence quenching dyes, and fiber-based excitation/collection forms the physical basis for the optical fiber DO sensors discussed in subsequent sections, which focus on their specific implementations and applications in bioprocess monitoring.

### 5.3. Advantages of Optical Fiber DO Sensors

Optical fiber DO sensors combine oxygen-sensitive luminescent chemistries with compact fiber-based optical readout, offering several advantages over conventional electrochemical probes in bioprocess monitoring. From a process perspective, they enable non-invasive and continuous measurement of DO with high sensitivity, can be miniaturized and integrated into a variety of culture formats, and are generally less prone to fouling and drift under complex bioreactor conditions. This subsection summarizes their main advantages in the context of modern bioprocesses.

#### 5.3.1. Real-Time, Continuous Monitoring

Because DO can change rapidly as cell density, oxygen uptake rate, and gas–liquid mass transfer conditions evolve, real-time, continuous monitoring is essential for detecting emerging oxygen limitation or oversupply and for maintaining process robustness.

Traditional electrochemical measurements are often performed intermittently or at a limited number of sampling points, which may miss short-term transients or local gradients [14,77]. In contrast, optical fiber DO sensors support real-time, continuous monitoring of DO without consuming oxygen during measurement, thereby providing an accurate picture of the dynamic oxygen environment experienced by the cells [76,77,78,79,80,81,82,83,84,85,86,87,88,89,90,91]. Continuous DO traces facilitate tighter feedback control of aeration, agitation, and pressure, and they are increasingly used in QbD and PAT frameworks to link oxygen availability to cell growth, metabolite formation, and critical quality attributes. This capability is particularly valuable in high-cell-density processes and perfusion systems, where DO can change rapidly and where early detection of oxygen limitation or oversupply is essential for maintaining process robustness.

#### 5.3.2. Progress in the Miniaturization and Integration of Sensing Technologies

A key advantage of optical fiber DO sensors lies in their small physical footprint and flexible integration options. Because the sensing head can be realized as a thin film on a fiber tip, a side-polished section, or a remote sensor patch read out through transparent walls, optical DO sensors can be embedded into shake flasks, single-use bags, microtiter plates, microbioreactors, and microfluidic devices without significantly disturbing the culture geometry [21,76,77,78,79,80,81,82,83,84,85,86,87,88,89,90,91]. Miniaturized parallel bioreactor systems with non-invasive optical DO sensors have been used for high-throughput strain screening and process optimization, enabling multi-point monitoring across small working volumes [80,81,82,83]. Microfluidic cell culture platforms and organ-on-chip devices have also benefited from integrated optical DO sensing, which allows spatially resolved measurements in channels and chambers that are inaccessible to conventional probes [72]. These developments illustrate how miniaturized optical DO sensors support scalable process development by bridging bench-scale, high-throughput, and production-relevant environments.

#### 5.3.3. High Sensitivity and Swift Response

Oxygen-sensitive luminophores such as PtOEP, PtTFPP, and ruthenium(II) complexes exhibit strong dynamic quenching behavior, which can be exploited to achieve high DO sensitivity over a broad concentration range [84,85,86,87,88,89,90,91]. As summarized in Table 5, reported I_0_/I ratios for optical DO sensors span approximately 1.3–117 depending on the dye–matrix combination and operating range, with platinum porphyrins in fluorinated polymers or core–shell silica nanoparticles showing particularly high sensitivities [84,85,88]. Response times can be tuned by selecting immobilization matrices with appropriate oxygen permeability and thickness; sensors based on PtOEP or Ru complexes in optimized sol–gel or polymer matrices often respond within tens of seconds or faster to changes between nitrogen and oxygen atmospheres [91]. For bioreactor applications, such a fast response enables timely detection of DO perturbations caused by feed changes, gas flow variations, or shifts in cellular oxygen demand, supporting rapid corrective actions and more stable process operation [76,77,78,79,80,81,82,83,84,85,86,87,88,89,90,91].

#### 5.3.4. Strong Contamination Resistance and Suitability for Complex Media

Compared with traditional electrochemical DO probes, which rely on membranes, electrolytes, and electrodes directly exposed to the culture medium, optical fiber DO sensors are generally more resistant to fouling and contamination in complex biological environments. The sensing chemistry is confined within a solid-state film or coating that is separated from the culture by a gas-permeable barrier, reducing susceptibility to biofilm formation, protein deposition, and cell adhesion [84,85,86,87,88,89]. Because the measurement principle is based on luminescence quenching rather than electrochemical reactions, there is no consumption of oxygen or generation of electrolysis products, and the sensors are less affected by medium composition, ionic strength, and redox-active species [84,85,86,87,88,89,90,91]. In disposable and single-use bioreactors, non-invasive optical DO patches read out by external fiber optics further minimize the risk of contamination associated with probe insertion and simplify sterilization and handling procedures [76,77,78,79,80,81,82,83].

### 5.4. Typical Application Cases of Optical Fiber DO Sensors

Optical fiber–based DO sensors have been implemented in a wide variety of bioreactor formats, ranging from shaken flasks and bench-scale stirred-tank reactors to parallel microbioreactors and microfluidic devices. Early studies demonstrated that luminescence-quenching probes and optical patches read out through fiber optics can provide reliable oxygen monitoring in shaken vessel systems that were previously limited to off-line measurements or indirect estimations. Subsequent work extended these concepts to stirred-tank bioreactors, high-throughput screening platforms, and organ-on-chip devices, highlighting the versatility of optical DO sensing for both microbial and mammalian cell cultures [72,73,74,75,76,77,78,79,80,81,82,83,84,85,86,87,88,89,90,91,92,93,94]. Representative implementations, including non-invasive shake-flask monitoring, continuous perfusion bioreactors, miniature stirred tanks, and microfluidic bioreactors, are summarized in Table 6.

Despite these advances, several challenges become evident when the published application cases are examined collectively. Many studies focus on proof-of-concept demonstrations under relatively simple conditions, with limited information on long-term stability, photobleaching, or sensor drift in real production environments. In shaken flasks and miniature bioreactors, sensor placement and local mixing behavior can lead to underestimation or overestimation of DO in poorly mixed regions, while in dense perfusion cultures, optical DO sensors may still struggle to capture steep spatial gradients near cell-retention devices or hollow-fiber modules [76,77,78,79,80,81,82,83]. In addition, calibration procedures are often tailored to specific media and operating conditions, which may limit the direct transfer of sensor calibrations between scales or different bioprocesses. These gaps underscore the need for more systematic evaluation of optical fiber DO sensors in long-duration, high–high-cell-density processes and under realistic industrial operating regimes.

## 6. Other Optical Fiber Sensor Applications in Bioreactors

In addition to pH and DO, a range of other process variables strongly influence bioreactor performance, including temperature, pressure, biomass concentration, and various metabolites. Optical fiber sensing technology provides a versatile platform that can be extended to these additional parameters by exploiting different optical mechanisms and functional materials.

### 6.1. Temperature Sensors

Temperature is another crucial parameter in bioprocessing, as it directly affects cell growth, metabolic activity, protein folding and aggregation, and ultimately the yield and quality of biotherapeutic products [7,14,52,53,54,55,95]. Even relatively small deviations from the optimal temperature range can trigger cellular stress responses, alter product glycosylation, or reduce viral and plasmid productivity. Accordingly, modern bioreactors rely on precise temperature monitoring and control to maintain stable culture conditions throughout the entire process.

Recent work has also explored high-pressure and high-temperature multi-parameter bioreactors that combine temperature monitoring with pressure, pH, and other critical variables. Vasile et al. developed a high-pressure/high-temperature bioreactor system with integrated sensors for microbial risk assessment in underground hydrogen storage, demonstrating stable temperature and pressure monitoring under extreme conditions [92]. Bannazadeh proposed a model predictive control strategy that jointly regulates DO and temperature during adeno-associated virus (AAV) production, highlighting the importance of accurate temperature measurements for advanced control in intensified bioprocesses [93]. At larger scales, Lee and co-workers reported a smart bioreactor equipped with fully integrated wireless multivariate sensors, including temperature, pH, DO, and glucose, for long-term in situ monitoring of stem cell cultures intended for clinical use [94]. These examples illustrate both the industrial demand for robust temperature control and the potential role of optical fiber temperature sensors in future multi-parameter monitoring architectures.

### 6.2. Pressure Sensors

Pressure is another vital parameter that requires monitoring in bioprocessing, particularly in gas–liquid systems where aeration, headspace pressure, and hydrodynamic conditions influence oxygen transfer, carbon dioxide stripping, and foam formation [7,14,52,53,54,55]. Excessive pressure can compromise vessel integrity, damage filters and single-use components, or impose mechanical stress on sensitive cell cultures, whereas insufficient pressure may reduce gas–liquid mass transfer and limit oxygen availability. Reliable pressure monitoring is therefore essential for maintaining safe operation and consistent process performance.

In bioreactor applications, pressure sensing based on optical fiber technology could help monitor bag integrity and structural loads in single-use systems, detect abnormal backpressure across spargers or filters, and support advanced control strategies that coordinate aeration, agitation, and off-gas management. When combined with temperature and DO monitoring, fiber-based pressure sensors would further enhance the ability to characterize and control the hydrodynamic environment experienced by cells [96,97]. However, dedicated studies on optical fiber pressure sensors in bioprocessing are still limited, and more work is needed to translate these concepts from structural and aerospace applications into validated, GMP-compliant bioreactor solutions.

### 6.3. Biomass Concentration Sensors

Apart from pH and DO levels, the concentration of viable biomass is a central indicator of culture performance and process state. Biomass concentration reflects cell growth kinetics, nutrient consumption, and metabolite accumulation, and it is often used to trigger feeding strategies, induction events, or harvest decisions [98,99]. Conventional biomass measurements rely on off-line techniques such as optical density (OD), dry cell weight, or automated cell counters, as well as soft sensors that infer biomass from off-gas analysis, metabolite profiles, or process models [100,101,102,103,104,105]. While these approaches can provide valuable information, they may lack spatial resolution, require manual sampling, or depend on empirical correlations that are not easily transferable between processes.

Optical fiber sensor technology offers new opportunities for biomass monitoring by exploiting changes in scattering, absorption, or intrinsic fluorescence of the culture. Fiber-based turbidity probes can be integrated into bioreactors to measure transmitted or backscattered light, providing continuous estimates of biomass concentration in situ [100,101,102,103,104,105]. In addition, optical fiber probes that monitor the autofluorescence of metabolic cofactors such as NADH and FAD can give insight into the metabolic state and viability of cells, complementing simple biomass measurements [9,18,22]. Although these approaches are less mature than pH and DO sensing, and calibration can be more complex due to the dependence on cell morphology, medium composition, and optical path length, they illustrate how optical fiber sensors can be extended beyond purely physicochemical parameters to capture biological state information.

For high-throughput and small-scale systems, miniaturized optical fiber biomass probes could be embedded in microtiter plates, shaken vessels, or microbioreactors, providing real-time cell density information without the need for frequent sampling. In larger bioreactors, combining biomass-sensitive scattering or fluorescence signals with distributed temperature and DO measurements would support more comprehensive process monitoring and model-based control strategies.

### 6.4. Monitoring Other Parameters

Beyond the mentioned parameters, optical fiber sensing technology has been applied or proposed for monitoring a range of additional variables relevant to bioprocessing, including glucose, lactate, carbon dioxide, specific metabolites, and even product-related quality attributes. Many of these sensors rely on immobilized enzymes, affinity ligands, or functional nanomaterials that convert the concentration of a target analyte into an optical signal, which is then interrogated by an optical fiber sensor. For example, plasmonic fiber probes and coated long-period gratings have been used to detect biomolecules and impurities via refractive index changes, while fluorescence- and absorbance-based fiber sensors have been developed for metabolites and small molecules in other application domains [18,19,20,33,34,35,36,39,40,41]. Although the temperature channel in this probe is not fiber-based, the design concept—co-locating temperature and chemical sensing elements in a compact, minimally invasive device—closely aligns with the multi-parameter optical fiber sensor systems envisioned for next-generation bioreactors [104].

In the context of bioprocessing, such optical fiber sensor designs could be adapted to monitor glucose and lactate as key indicators of cellular metabolism, measure dissolved carbon dioxide alongside pH and DO, or track product titer and aggregation state in real time. Multi-parameter optical fiber sensor platforms that combine pH, DO, temperature, and one or more metabolite channels along a single fiber would be particularly attractive for intensifying upstream and downstream operations. However, most reported optical fiber sensors for these additional parameters have not yet been fully validated in industrial bioreactors, and issues such as coating stability, biofouling in real cell culture media, and regulatory acceptance remain to be systematically addressed.

These examples illustrate that optical fiber sensors can be extended beyond pH and DO to temperature, pressure, biomass, and other analytes, using many of the same fiber architectures and interrogation concepts. When integrated into standardized, automation-ready bioreactor platforms and combined with advanced data analytics and control strategies, these multi-parameter optical fiber sensor systems are expected to play an increasingly important role in the bioprocessing sector, enriching monitoring and control capabilities and supporting the implementation of PAT and QbD principles in both development and manufacturing.

## 7. Challenges and Future Prospects

In the preceding sections, we have summarized recent progress in applying optical fiber sensors to monitor key bioprocess parameters, including pH, DO, temperature, pressure, biomass, and selected metabolites. These studies demonstrate that fiber-based approaches can deliver compact, minimally invasive, and highly adaptable monitoring solutions across scales ranging from shake flasks and microreactors to single-use bags and stainless-steel bioreactors. At the same time, they reveal several technical and practical limitations that currently confine many optical fiber sensors to laboratory demonstrations rather than routine industrial use. Addressing these limitations defines clear directions for future research and development.

### 7.1. Key Challenges

#### 7.1.1. Sensor Robustness and Coating Stability in Real Bioprocess Media

Many optical fiber sensors discussed in this review rely on functional coatings, such as sol–gel layers, hydrogels, polymer matrices, and nanoparticle- or dye-doped films. While these materials enable high sensitivity and tunable selectivity, their long-term stability in real cell culture media remains a major challenge. Swelling, leaching, photobleaching, biofouling, and mechanical delamination can gradually degrade sensor performance during extended runs, repeated sterilization cycles, or cleaning-in-place procedures [106,107,108,109,110]. In addition, practical devices must withstand storage, shipping, and sterilization before use. Systematic studies of coating durability under realistic bioprocess conditions, development of more robust encapsulation and surface treatments, and the use of reference channels or redundancy to detect gradual drift will be essential to move from proof-of-concept demonstrations to reliable, GMP-compatible monitoring tools.

#### 7.1.2. Calibration, Cross-Sensitivities, and Standardization

Optical fiber sensors are often intrinsically multi-parameter sensitive: temperature, strain, refractive index, and analyte concentration can all influence the measured wavelength or intensity [109,110,111,112,113,114,115]. This creates cross-sensitivities that complicate calibration, especially in complex cell culture media where several variables change simultaneously [111,112,113]. In many published studies, calibration is performed under simplified buffer conditions and may not fully represent real bioprocess environments, which makes it difficult to compare performance claims across different sensor designs and suppliers. Moving toward practical deployment will require multi-parameter calibration strategies that explicitly account for temperature and matrix effects, the use of internal references or compensation schemes, and the development of standardized test protocols and reporting formats so that sensor performance can be benchmarked in a consistent and transparent way [114].

#### 7.1.3. Mechanical Integration with Stainless-Steel and Single-Use Bioreactors

Integrating fragile glass fibers and miniature sensor heads into bioreactor hardware is non-trivial. In stainless-steel systems, optical feedthroughs and sensor ports must maintain sterility and withstand pressure, temperature, and repeated cleaning cycles, while protecting the fibers from bending and mechanical damage. In single-use bioreactors, bag films and port designs were originally optimized for electrochemical probes and fluid connections, not for embedded optical fibers [115,116]. Routing fibers through films or sealing them into pre-sterilized ports without compromising bag integrity, gas tightness, and robustness remains a design challenge. Similar constraints appear in shake flasks, microtiter plates, microfluidic devices, and microbioreactors, where space is limited and mechanical agitation or flow may induce additional stress on the fibers.

#### 7.1.4. Data Volume, Signal Processing, and Control Integration

Many optical fiber sensor configurations, especially multiplexed and distributed systems, generate large volumes of spectral or time-resolved data. Extracting reliable physical parameters from these signals requires robust algorithms for wavelength demodulation, phase or intensity referencing, drift compensation, and noise filtering. When multiple sensing points or parameters are monitored simultaneously, real-time data processing becomes more demanding. Integrating these data streams into existing control architectures and PAT frameworks and ensuring that derived soft sensors or AI-assisted models are transparent and validated is another challenge [116]. Without user-friendly software and standardized data interfaces, the barrier to adoption in routine manufacturing will remain high.

#### 7.1.5. Cost, Complexity, and Required Expertise

Although the cost of optical components has decreased, industrial-grade interrogators, lasers, and spectrometers are still relatively expensive compared with conventional electrochemical pH and DO probes [100,101,102,103,104,105,106,107,108,109,110,111,112,113,114,115,116]. In addition, the design, fabrication, and maintenance of optical fiber sensors often require specialized photonics expertise that may not be readily available in bioprocess development teams. For many organizations, the perceived complexity of optical systems, combined with limited internal experience, discourages early adoption. Demonstrating clear cost–benefit ratios—such as improved process understanding, reduced failure risk, or increased product quality—is essential to justify investment in optical fiber sensing platforms.

#### 7.1.6. Regulatory and Validation Barriers

Biopharmaceutical manufacturing is tightly regulated, and any new measurement technology must pass through rigorous qualification, validation, and lifecycle management. Regulators and quality organizations are familiar with the capabilities and failure modes of traditional pH and DO probes, but have less experience with optical fiber sensors, especially multi-parameter and distributed configurations. Comprehensive comparability studies against existing technologies, clear documentation of accuracy, precision, robustness, and failure behavior, and well-defined calibration and maintenance strategies are needed before optical fiber sensors can be fully accepted as primary or even secondary PAT tools in GMP environments. Until such evidence accumulates, many implementations will remain confined to development laboratories or non-critical monitoring roles.

### 7.2. Future Prospects and Research Directions

Despite these challenges, the outlook for optical fiber sensing in bioprocessing is highly promising. Recent advances in materials, fiber designs, interrogation techniques, and data analytics are converging with industry trends towards intensified, automated, and data-rich manufacturing. Several research directions appear particularly impactful.

#### 7.2.1. Multi-Parameter and Distributed Sensing Architectures

One major opportunity lies in the design of multi-parameter optical fiber sensors that simultaneously monitor pH, DO, temperature, pressure, glucose, lactate, and possibly biomass along the same fiber. Combining different sensing mechanisms—such as long-period gratings, tilted fiber Bragg gratings, Fabry–Pérot cavities, and surface plasmon resonance structures—on a single fiber could enable dense multiplexing of localized sensing sites. In large stainless-steel vessels and high-value single-use reactors, distributed sensing techniques based on OFDR, Raman, or Brillouin scattering may provide spatially resolved temperature or strain information that complements localized chemical measurements [24,25,28,30,32]. These architectures could capture gradients and heterogeneities that are invisible to single-point probes and are highly relevant for scale-up and continuous bioprocessing.

#### 7.2.2. Advanced Functional Materials and Packaging Strategies

Continued development of robust functional coatings is crucial. Promising directions include hydrogels and sol–gel matrices with reduced swelling and improved fouling resistance, fluorinated or ORMOSIL polymers with high oxygen permeability and chemical stability, and nanostructured or plasmonic films that enhance sensitivity while protecting the underlying fiber [109,110,111,112,113,114,115]. At the same time, mechanical packaging concepts—such as sensor patches, protective capillaries, 3D-printed housing, and plug-and-play connectors for bags and reactors—will be needed to translate delicate lab prototypes into industrially deployable devices [73]. Modular packaging that is compatible with current single-use hardware standards would greatly facilitate adoption [99].

#### 7.2.3. Integration with PAT, Soft Sensors, and AI-Driven Control

Optical fiber sensors are naturally aligned with the goals of PAT and QbD, as they provide real-time, in situ information on critical process parameters and, potentially, critical quality attributes. In the future, fiber-based measurements will increasingly be combined with mechanistic models, data-driven soft sensors, and AI/ML tools to estimate hard-to-measure variables, detect anomalies, and support advanced control strategies. For example, high-frequency pH, DO, and temperature profiles from optical fiber sensors could be fused with off-gas analysis, Raman spectra, and process models to build digital twins of bioreactors. These digital twins could then be used to optimize feed strategies, predict batch outcomes, and enable real-time release testing.

#### 7.2.4. Standardization, Benchmarking, and Regulatory Engagement

To move from promising demonstrations to widespread industrial use, the community needs coordinated efforts in standardization and benchmarking. This includes developing reference test protocols for accuracy, response time, stability, and fouling resistance in representative media; organizing inter-laboratory comparison studies; and defining metrics that allow fair comparison between different sensor designs and suppliers. Early engagement with regulatory agencies and industry consortia can help shape guidelines for validation and lifecycle management of optical fiber sensors, reducing uncertainty for adopters. Transparent reporting of performance limitations and failure modes will also build confidence in these technologies.

#### 7.2.5. Application Expansion Across Scales and Modalities

Finally, optical fiber sensing is expected to play an important role in bridging the gap between small-scale development and large-scale manufacturing. In early-stage screening, miniaturized fiber sensors integrated into microtiter plates, shaken systems, and microbioreactors can provide richer data for clone selection and process optimization. At pilot and production scales, robust multi-parameter fiber sensors in stainless-steel and single-use reactors can ensure that optimized conditions are faithfully reproduced, while distributed sensing may reveal scale-dependent heterogeneities. Beyond conventional CHO cell processes, similar concepts can be extended to stem cell cultures, viral vector production, microbial fermentation, and continuous bioprocessing platforms.

## 8. Conclusions

Optical fiber sensing has emerged as a powerful and highly adaptable platform for monitoring key parameters in bioprocessing. This review has summarized recent progress in applying optical fiber sensors to pH and DO as core critical process parameters and has outlined extensions to temperature, pressure, biomass, and selected metabolites. By revisiting conventional monitoring tools and their limitations, introducing the main optical fiber sensing mechanisms and architectures, and comparing representative implementations in bioreactors, we have highlighted how fiber-based approaches can provide compact, minimally invasive, and scalable solutions across shake flasks, single-use systems, and stainless-steel reactors.

Despite these advances, several challenges still limit widespread industrial adoption. Long-term stability of coatings and packaging in real culture media, cross-sensitivities among temperature, refractive index, and chemical composition, and the lack of harmonized calibration and validation protocols remain critical issues. Practical integration into existing bioreactor hardware, user-friendly interrogation and data processing, and alignment with regulatory expectations must also be addressed. Looking ahead, the most promising directions combine robust multi-parameter and distributed fiber architectures with advanced signal processing, soft sensors, and AI-enabled control within PAT and QbD frameworks. As these technical and regulatory barriers are progressively overcome, optical fiber sensors are expected to play an increasingly central role in more intensified, automated, and data-driven biomanufacturing.

## Figures and Tables

**Table 1:** Highlights bioprocess-specific pH and DO sensor requirements that extend beyond standard expectations, such as analytical performance, calibration needs, robustness, stability, user handling, compatibility, validation ease, and timely response.

Category	Growth Characteristics	Typical Doubling Time	Cultivation Duration	Operational pH Range	Typical DO Range
**Microorganisms** *Escherichia coli* *Pichia pastoris*	Primarily cultivated as suspension cultures. Capable of reaching medium to very high biomass levels. Culture fluids may behave as Newtonian or non-Newtonian depending on density.	Minutes	Days	2–12	20–60%
**Plant cells** *Taxus baccata*, *Daucus carota*	Grown as suspension cultures or hairy-root systems. Exhibit low to moderate biomass accumulation. Broth rheology varies with cell density.	Days to weeks	Weeks to months	5–6	
**Mammalian cells** Chinese hamster ovary HEK293	Mainly suspension cultures. Adherent two- or three-dimensional systems. Middle to high cell densities. Newtonian culture broths.	Hours to days	Days to weeks	6.8–7.4	
**Insect cells** *Spodoptera frugiperda*, High-five cell lines			Days	6.1–6.5	
**Stem cells** Mesenchymal stem cells from bone marrow or adipose tissue	Adherent two- and three-dimensional cultures. Generally maintained at low densities. Newtonian culture broths.			6.8–7.4	0.7–20%

**Table 2:** Classification of traditional pH and DO monitoring methods in bioprocessing.

Monitoring Category	Typical Principles/Tools	Measurement Mode	Main Advantages	Key Limitations	Typical Use/Scale
Inline electrochemical probes	Glass pH electrodes; polarographic or galvanic DO probes mounted in the bioreactor headplate or side ports	Continuous, inline	Real-time signal for feedback control; mature technology; directly integrated with standard bioreactor controllers	Susceptible to drift, fouling, and environmental interference; single-point measurement; requires regular calibration and maintenance	Laboratory and industrial stirred-tank and airlift bioreactors from bench to production scale
Off-line and at-line analytical instruments	Benchtop pH meters; blood-gas analyzers; biochemical analyzers measuring pH, DO, and related variables	Discrete, sampling-based	High analytical accuracy under controlled conditions; can measure additional parameters; independent verification of inline probes.	Time delay between sampling and result; additional handling and contamination risk; labor-intensive and difficult to automate fully	Process development laboratories and GMP environments for method validation and quality control
Single-use and miniaturized electrochemical probes	Pre-mounted pH and DO sensors in disposable bioreactor bags; miniature probes integrated into small-scale or high-throughput systems	Continuous or quasi-continuous, inline or in situ	Compatible with disposable systems and parallel small-scale reactors; low working volume; easier deployment in high-throughput studies	Limited lifetime and sterilization options; calibration and standardization can be challenging; sensor performance may vary between batches	Single-use bioreactors, microbioreactors, and scale-down models for process development

**Table 3:** Main categories of optical fiber sensing mechanisms relevant to pH, DO, and related bioprocess parameters, with typical fiber configurations, encoded optical quantities, and qualitative advantages and limitations.

Sensing Mechanism	Typical Fiber Structure/Configuration	Encoded Optical Quantity	Main Advantages	Typical Limitations
Intensity-based	Straight or tapered fiber; side-polished fiber; dye- or indicator-coated fiber tip or segment	Transmitted, reflected, or fluorescence intensity	Simple design and readout; low-cost hardware; easy integration and miniaturization	Sensitive to source and coupling fluctuations; bending and loss variations can affect the signal
Wavelength-encoded	Fiber Bragg gratings (FBGs); long-period fiber gratings (LPFGs); Fabry–Pérot cavities; multimode interferometers; plasmonic structures	Resonance or fringe wavelength	High resolution; robust against power drift; suitable for multiplexing and multi-parameter measurement	Requires a spectrometer or wavelength-resolved interrogation; more complex alignment and signal processing.
Phase-based	Mach–Zehnder, Michelson, or Sagnac interferometers; dual-core or multicore fibers	Phase shift or fringe position	Very high sensitivity to small refractive index or path-length changes; compatible with dynamic measurements	Interrogation and stabilization can be complex, sensitive to environmental perturbations (vibration, temperature)
Polarization-based	Polarization-maintaining fibers; birefringent fiber structures; fiber loop mirrors	Polarization state, birefringence, or beat length	Good sensitivity to stress, temperature, and anisotropic changes; useful for vector quantities	Requires polarization control and analysis; prone to random polarization fluctuations in non-PM fibers

**Table 4:** Representative bioreactor pH monitoring applications using optical fiber sensors with key performance metrics.

Year	Authors	Sensing Mechanism Used	Important Results	Key Performance	Reference
1993	Holobar et al.	Optical	Non-invasive pH measurement system.	pH range: 6–10. Accuracy: ±0.1.	[59]
2002	Kermis et al.	Dual Excitation Ratiometric Fluorescent	Non-invasive bioprocess monitoring.	pH range: 6–9. Accuracy: ±0.05.	[49]
2003	Kermis et al.	Optical	Rapid method development for robust pH sensors.	pH range: 6–9.	[50]
2004	Weidgans et al.	Fluorescent	pH sensors with minimized sensitivity to ionic strength variations.	pH range: 4.5–8.	[51]
2011	Shen et al.	Long-wavelength Fluorescent Hydrophilic Copolymer	Broad linear response range, enhancing pH monitoring accuracy.	pH range: 4.6–8.	[54]
2016	Janzen et al.	Fluorescent	Suitable for monitoring at low pH in challenging fermentation processes.	pH range: 3.9–7.2.	[55]
2016	Mousavi Shaegh et al.	Microfluidic Optical	Real-time monitoring for microfluidic bioreactors and organ-on-chip devices.	Measure continuously for up to 3 days.	[56]
2016	Demuth et al.	Novel optical Probes	Challenges and solutions for pH sensing across scales.	N/A	[52]
2020	Newton et al.	Fluorescent	Process-adapted calibration method for improved accuracy in complex fermentation.	pH range: 6–8. Accuracy: ±0.1.	[57]
2022	Udomsom et al.	Automatic optical Programmable	Real-time pH monitoring for tissue engineering applications.	pH range: 6.5–8.	[53]

**Table 5:** Overview of key performance parameters for optical DO sensors by dye family and immobilization matrix.

Dye Family	Representative Immobilization Matrices	Typical Response Characteristics	Sensitivity	Reference
(PtOEP, PtTFPP)	Fluorinated co-polymers; TEOS/C_8_TEOS; core–shell silica nanoparticles	Fast to moderate, depending on the matrix; nanoparticle systems facilitate improved O_2_ diffusion	I_0_/I ≈ 1.8 to >100 (0–40 mg/L)	[84,85]
Ru (Ph_2_phen)_3_^2+^	Fluoropolymer coatings; TEOS/MTEOS sol–gel matrices	Generally rapid response; suitable for low-to-mid oxygen levels	I_0_/I ≈ 3–6.6 (0–40 ppm)	[86,87]
Ru (dpp)_3_^2+^	TMOS/DiMe-DMOS; TMOS/C_8_TMOS; TEOS/MTEOS	Moderate switching kinetics (≈20–100 s depending on O_2_/N_2_ transition)	I_0_/I ≈ 1.3–16 (0–100% O_2_)	[88,91]
(Ru(bpy)_3_^2+^)	Silica–Ni–P composites; TMOS/DiMe-DMOS sol–gels	Slower response (≈66–300 s)	I_0_/I ≈ 2.6–7.3 (0–20 × 10^−6^ M)	[89,90]

**Table 6:** Representative bioreactor DO monitoring applications using optical DO sensors with key performance metrics.

Year	Authors	Sensing Mechanism Used	Important Results	Key Performance	Reference
2000	Kostov, Yordan, et al.	Dual emission probe and solid-state ratiometric fluorometer	Unique oxygen analyzer development	DO range: 0–90%.	[81]
2002	Tolosa, Leah, et al.	Noninvasive optical	Introduced a method for noninvasive DO measurement in shake flasks	DO range: 0–60%.	[82]
2003	Gupta, Atul, and Govind Rao	Noninvasive optical	Study on oxygen transfer in shake flasks	DO range: 0–60%.	[83]
2004	Gillanders et al.	PtOEP (fluorinated co-polymer)	Enabled efficient oxygen monitoring in continuous cell culture	DO range: 0–100%. Response time: 10 s.	[84]
2005	Gillanders et al.	Fluorescence quenching of Ru(II) complex immobilized in blended fluoropolymer film	Developed an optical fiber sensor	DO range: 0–30 mg/L. Sensitivity: 0.1 mg/L.	[85]
2006	Tao et al.	Class II xerogel-based optical O_2_ sensors (tunable sensitivity)	Evaluated the oxygen transfer performance of miniature bioreactor platforms	DO range: 0–100%.	[88]
2015	Chu et al.	PdTFPP/CdSe embedded in sol–gel	Optical fiber sensor development for dual sensing	DO range: 0–40 mg/L.	[90]
2022	Zhao et al.	Ratiometric optical fiber DO sensor (Ru(dpp)_3_^2+^ + QDs)	Reported a ratiometric optical fiber DO sensor with linear Stern–Volmer behavior.	DO range: 0–18.25 mg/L.	[77]
2024	Lee et al.	Integrated multivariate sensor array in large-scale bioreactor	Large-scale smart bioreactor enabling multi-spatial sensing (pH, glucose, DO, temperature) for long-term culture monitoring.	DO range: 0–115 mg/L. Measure continuously for up to 30 days.	[94]

## Data Availability

No new data were created or analyzed in this study. Data sharing is not applicable to this article.
